# Adverse drug reaction monitoring with angiotensin converting enzyme inhibitors: A prospective, randomized, open-label, comparative study

**DOI:** 10.4103/0253-7613.62408

**Published:** 2010-02

**Authors:** Nishant V. Sangole, Vaishali N. Dadkar

**Affiliations:** Department of Pharmacology, Pad. Dr. D.Y. Patil Medical College, Hospital and Research Center, Nerul, Navi Mumbai, India

**Keywords:** Angiotensin converting enzyme inhibitor, adverse drug reaction, angiotensin converting enzyme inhibitors, antihypertensive, enalapril, fosinopril, lisinopril, ramipril, dicarboxyl, phosphonate

## Abstract

**Objectives:**

Angiotensin converting enzyme inhibitors (ACEIs) are known to possess different chemical structures, and change in structure of a drug can bring about change in its adverse drug reaction (ADR) profile. The study aims to observe the incidence and severity of ADRs between the di-carboxyl group containing ACEIs (d-ACEIs) versus phosphonate group containing ACEIs (p-ACEIs), in patients suffering from essential hypertension.

**Materials and Methods:**

One hundred and twenty patients with essential hypertension were randomized into four groups receiving enalapril, lisinopril, ramipril, and fosinopril. They were followed up for four months, to observe the clinical efficacy along with the associated ADRs.

**Results:**

Mild, dry brassy cough (% incidence; 95% CI) was observed with d-ACEIs (6.6%; 0 to 15.6) versus p-ACEI (20%; 5.7 to 34.3), in which the cough observed was moderate-to-severe in intensity and two patients required treatment discontinuation (*P* < 0.05). No cases of hypotension were observed with the use of d-ACEIs, whereas, two patients on p-ACEI (6.6%; 0 to15.6) had hypotension (*P* < 0.05). Three patients (10%; 0 to 20.7) on d-ACEIs had nausea, which was not observed with p-ACEI treatment (0%) (*P* < 0.05).

**Conclusions:**

The phosphonate group in p-ACEIs may have a probable relationship with increase in the incidence and severity of ADRs such as dry brassy cough and hypotension. The di-carboxyl group in d-ACEIs may have a probable relationship with increase in the incidence of ADRs like nausea.

## Introduction

Angiotensin converting enzyme inhibitors (ACEIs) have a well-established role in the management of essential hypertension. They are structurally classified as sulfhydryl containing ACEIs, for example, captopril, fentiapril, zofenopril, and so on; di-carboxyl containing ACEIs namely enalapril, lisinopril, perindopril, quinapril, moexipril, and so on; and phosphonate containing ACEIs namely fosinopril, on the basis of their binding with the angiotensin converting enzyme (ACE).[1] It is a well-known fact, that the activity of a drug can be modified by changes in the structure of the drug. Hence, it can be hypothesized that they may have different patterns of adverse drug reactions (ADRs). This study was prompted by the fact that a large number of people suffer from essential hypertension and ACEIs certainly are among the most widely prescribed agents in its treatment. It is therefore imperative that we should have maximum data on their pattern of utilization and the adverse drug reactions.

The purpose of the present study was to observe the incidence and severity of adverse drug reactions between the di-carboxyl group containing ACE inhibitors (namely enalapril, lisinopril, and ramipril) versus the phosphonate group containing ACE inhibitors (namely fosinopril), in patients suffering from essential hypertension.

## Materials and Methods

One hundred and twenty newly diagnosed patients (83 male, 37 female) suffering from stage I / II essential hypertension, according to JNC-VII guidelines,[2] without any underlying comorbid conditions or complications, aged between 20 and 70 years, were enrolled in the study after obtaining informed consent and due approval of the ethics committee. It was a prospective, parallel, open-label, randomized, comparative trial, and the patients were divided into four groups of 30 each. Each group received enalapril (2.5 mg), lisinopril (2.5 mg), ramipril (2.5 mg), and fosinopril (10 mg), respectively, once daily, in the morning with breakfast. As it was an open-label study, to avoid selection bias, a randomization chart was prepared beforehand using the Random Allocation Software (version 1.0.0; developed by M.Saghaei and Isfahan). In this randomization chart, the subject numbers were allocated from 1 – 160 (anticipating study dropouts) in four groups. Each subject was enrolled on a first-come basis and received treatment as per his / her randomization number. The investigational drugs were prescribed to the study subjects and purchased from the hospital pharmacy. The individual dose was subsequently titrated in case of inadequate blood pressure control, which was predefined for blood pressure levels of < 140 / 90 mmHg.

On confirming the diagnosis, the baseline blood pressure in the left arm (sitting position) was recorded after allowing 10 minutes of rest for each subject. The blood pressure of all the subjects was monitored on an hourly basis, for four hours on day 1, after the first dose was administered, under supervision. Every subject was followed up for four months, which included eight follow-ups at an interval of 15 days. During every follow-up, the blood pressure in the left arm (sitting position) was recorded after allowing 10 minutes of rest, the compliance with therapy and use of concomitant medicines was documented; any additional anti-hypertensive medication precluded the subject from continuing in the study. Hematological and biochemical examinations were performed at baseline and end of the study. Adverse Events (AEs) if any, were documented during the follow-up visit and their causality was assessed using the World Health Organization-Uppsala Monitoring Center (WHO-UMC) causality assessment criteria.[3] For grading adverse drug reactions, such as, dizziness, cough, musculoskeletal pain, fatigue, headache, and nausea, a visual rating scale (VRS) was employed; cough was further evaluated on the basis of its interference in routine activities and sleep disturbances in the subject. To propose a hypothesis, after comparing the incidence of ADRs between the two groups, namely di-carboxyl containing ACE Inhibitors and phosphonate containing ACE Inhibitors, we employed the statistical hypothesis test of difference in proportion (Z-test), to calculate the P-value in terms of significance.

## Results

**Figure 1:**
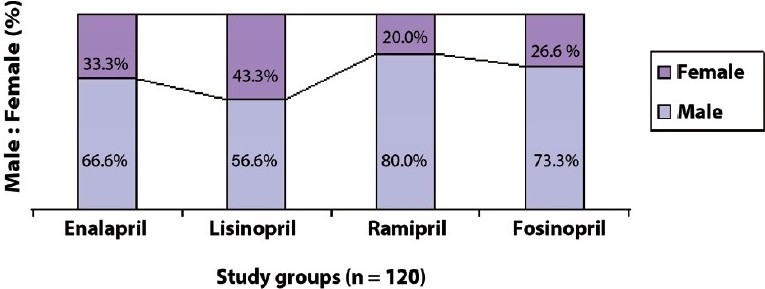
Gender distribution of patients receiving ACEI for hypertension

The unicentric, prospective, randomized, comparative, open-label study included 120 patients suffering from stage I / II essential hypertension. These included 83 males and 37 females randomized in each study group [Figure 1]. It was evident that the number of males in each study group was more than the females. Their mean (± S.D.) age was 50.7 (± 16.9) years; baseline blood pressure (systolic / diastolic), 157.4 (±15.2) / 101.4 (±9.1) mm Hg; and body mass index, 24.0 (±2.5) kg/m^2^. The target blood pressure of ≤ 140/ 90 mm Hg was achieved in all subjects by appropriate individualized dose titration. The mean (±SD) blood pressure at end of the study was observed as 129.6 (±10.2) / 89.4 (±7.1) mm Hg. The study drugs were tolerated by the majority, except in five subjects, who presented with adverse events such as neutropenia (n = 1), severe dry cough (n = 2), and skin rash (n = 2), which warranted treatment discontinuation. During the course of the study five subjects dropped out, of which four subjects were withdrawn due to lack of three consecutive follow-up visits and one subject dropped out, as the subject could not bear the expenses involved. New cases were enrolled to compensate the dropouts.

**Figure 2:**
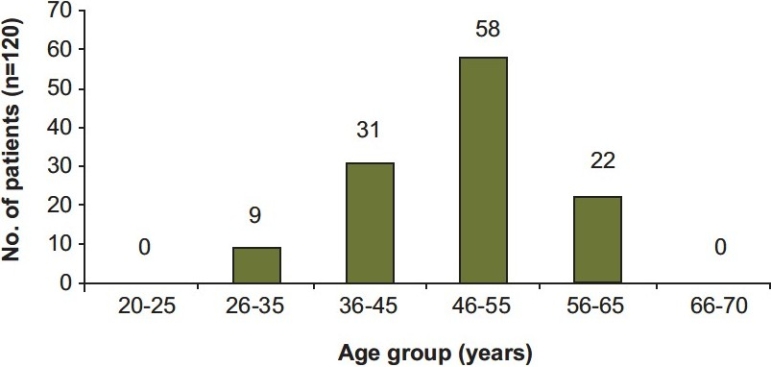
Age distribution of study subjects receiving ACEI for hypertension

The observed age distribution of subjects receiving ACEIs in each study group is expressed in Figure 2. It is evident that a majority (48.3%) of the subjects were in the age range of 46 – 55 years, whereas, only 7.5% of the population was in the age group of 26 – 35 years.

The various adverse drug reactions observed in the study subjects were dizziness, cough, hypotension, diarrhea, hyperkalemia, neutropenia, musculoskeletal pain, fatigue, headache, nausea, and skin rash [Table 1].

As evident from the findings [Table 1], significant differences with regard to the incidence and severity were observed only with cough, hypotension, and nausea.

**Table 1:** Summary of incidence of all adverse drug reactions observed in the study subjects (n = 120)

*Observed adverse drug reactions*	*Enalapril n = 30 total number (%)*	*Lisinopril n = 30 total number (%)*	*Ramipril n = 30 total number (%)*	*Fosinopril n = 30 total number (%)*
Dizziness	2 (6.6%)	4 (13.3%)	1 (3.3%)	4 (13.3%)
Cough*	2 (6.6%)*	2 (6.6%)*	2 (6.6%)*	6 (20%)*
Hypotension*	0*	0*	0*	2 (6.6%)*
Diarrhea	0	2 (6.6%)	0	1 (3.3%)
Hyperkalemia	0	3 (10%)	2 (6.6%)	0
Neutropenia	1 (3.3%)	0	0	0
Musculoskeletal	1 (3.3%)	1 (3.3%)	0	2 (6.6%)
pain				
Fatigue	1 (3.3%)	2 (6.6%)	0	2 (6.6%
Headache	2 (6.6%)	0	1 (3.3%)	0
Nausea*	3 (10%)*	3 (10%)*	3 (10%)*	0*
Skin Rash	1 (3.3%)	1 (3.3%)	0	0

* Indicates *P* < 0.05, 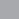 Dicarboxyl group containing ACEIs, 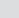 Phosphonate group containing ACEI

### Cough

Two subjects (%incidence; 95%CI) on enalapril (6.6%; 0 to15.6), two subjects on lisinopril (6.6%; 0 to 15.6), two subjects on ramipril (6.6%; 0 to 15.6), and six subjects on fosinopril (20%; 5.7 to 34.3) had dry cough. A total of 12 cases (nine males and three females) of cough were observed [Table 1]. All the subjects gave a history of dry, non-productive cough. Details regarding the intensity of the cough and other related features are tabulated in Table 2.

**Table 2:** Cough seen in study subjects receiving the ACEIs for hypertension (n = 12).

*Drug*	*No. of cases*	*Sex distribution (M-Male and F- Female)*	*Onset*	*Nature*	*Discontinuation from therapy*	*Sleep disturbances*
Enalapril	2	1 M + 1 F	1 month	mild	No	No
Lisinopril	2	1 M + 1 F	1 ½ months	mild	No	No
Ramipril	2	2 M + 0 F	1 – 1 ½ months	mild	No	No
Fosinopril	6	5 M +1 F	15 – 20 days	moderate-to-severe	2 subjects	Yes

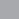
 Dicarboxyl group containing ACEIs, 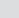 Phosphonate group containing ACEI, Comparing the two groups, *P* < 0.05 using one tailed P-value.

Subjects receiving enalapril, lisinopril, and ramipril (the d-ACEIs) developed dry cough after one to one-and-a-half months of therapy. In all these subjects, the cough was mild in nature and there were no specific aggravating or relieving factors. The cough continued throughout the period of observation in all the subjects and did not cause any disturbances in their routine activities or any kind of sleep disturbances. It did not warrant discontinuation of therapy.

On the other hand, six subjects (five males and one female) receiving fosinopril (belonging to p-ACEI) presented with dry cough within fiftten days of initiating therapy in four subjects and within twenty days of initiating therapy in two subjects. The cough worsened at night, in the lying down position, in all the six subjects. It was progressive from mild-to-moderate in four subjects, but did not interfere in their routine activities. Two subjects were discontinued from fosinopril and started on another anti-hypertensive agent. The cough subsided on the seventh day after discontinuation of fosinopril.

The overall incidence of cough appeared to be higher in males compared to females. This could be attributed to the fact that the number of males enrolled in this study was higher.

### Hypotension

There were no cases of hypotension observed with enalapril (0%), lisinopril (0%) or ramipril (0%). Although, two subjects (%incidence; 95%C.I.) on fosinopril (6.6%; 0 to 15.6) had hypotension; one among them gave a history of associated dizziness [Table 3].

**Table 3:** Hypotension seen in study subjects receiving ACEI for hypertension (n = 2).

*Drug*	*Mean B.P.(baseline) mmHg*	*Mean B.P.(After 1 month of therapy) mmHg*	*Dose range*	*Cases of hypotension*
Enalapril	142/98	128/88	2.5 – 5 mg	0
Lisinopril	140/100	126/88	2.5 – 5 mg	0
Ramipril	146/94	128/86	2.5 – 5 mg	0
Fosinopril	144/100	118/82	10 – 20 mg	2

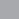
 Dicarboxyl group containing ACEIs, 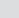 Phosphonate group containing ACEI Comparing the two groups, *P* <0.05 using one tailed P-value.

### Nausea

Three subjects (%incidence; 95%C.I.) on enalapril (10%; 0 to 20.7), three subjects on lisinopril (10%; 0 to 20.7), and three subjects on ramipril (10%; 0 to 20.7), all belonging to d-ACEIs, presented with nausea. The nausea was mild-to-moderate in intensity. The time of onset was 40 – 80 minutes after consuming the drug and it lasted for another two to three hours in all the subjects. There were no associated episodes of vomiting. Nausea did not warrant discontinuation of therapy. On the other hand, not a single case of nausea was reported with fosinopril (0%). A summary of these features is presented in Table 4.

**Table 4:** Nausea observed in study subjects receiving ACEI for hypertension (n = 9)

*Drug*	*No. of cases*	*Intensity*	*Time of onset(minutes)*	*Duration*
Enalapril	3	mild	40 – 45	2 – 3
Lisinopril	3	mild-to-moderate	60 – 80	2 – 3
Ramipril	3	mild-to-moderate	45 – 60	2 – 3
Fosinopril	0	-	-	-

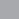
 Dicarboxyl group containing ACEIs, 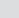 Phosphonate group containing ACEI Comparing the two groups, *P* <0.05 using one tailed P-value.

Among the various adverse events reported in this study, every AE remained independent of one another and there was no overlapping observed; except in one subject, who presented with hypotension and dizziness.

## Discussion

Besides essential hypertension, ACEIs are increasingly used for the management of several other conditions, such as, acute myocardial infarction, left ventricular systolic dysfunction, chronic renal failure, and so on. Large multi-centric trials[4,5] have proved that ACEIs not only increase the life expectancy, but also improve the quality of life in high-risk patients suffering from cardiovascular events. It appears that by their specific effect on myocardial and vascular cell growth, also referred to as remodeling, they have a greater protective potential than any other class of anti-hypertensive drugs.

Ever since captopril (sulfhydryl group containing ACEI) was introduced as an anti-hypertensive agent in the year 1981, by the USFDA, many adverse effects with its use have been reported. Several other ACEIs followed captopril over the years to come and today we have a surfeit of ACEIs to choose from with more or less a similar ADR profile.

Our study was designed to monitor the various ADRs seen with the ACEIs containing the di-carboxyl group (namely enalapril, lisinopril, and ramipril) and the phosphonate group (namely fosinopril), with the aim to observe the incidence and severity of ADRs between the two groups.

Among the 11 ADRs [Table 1] that were encountered with various ACEIs, only three ADRs namely cough, hypotension, and nausea elicited a significant difference related to their chemical structure. The incidence of these ADRs was not related to the age group of the study subjects.

Dry, brassy cough is commonly reported with the use of ACEIs and is estimated to be in the range of 5-10%.[6–9] The cough is usually persistent, paroxysmal, non-productive, worsening in the lying down position, and at times accompanied by a change in voice.[10] Studies have suggested the involvement of mediators such as, bradykinin, prostaglandins or substance P as mediators of the cough.[11,12] A literature survey suggests about a 6% incidence of cough with enalapril, lisinopril, and ramipril,[6–8] whereas, it is reported to be higher (10%) with fosinopril.[9]

In our study, the incidence of cough with enalapril, lisinopril, and ramipril was similar to that reported in literature, but was distinctly higher (20%) in subjects receiving fosinopril. It was interesting to note that some of the subjects (three males and one female) on fosinopril also complained of sleep disturbances on account of cough, of which two (one male and one female) required discontinuation of drug as cough was very troublesome. This could have possible repercussions on the long-term use of p-ACEIs. Hence, d-ACEIs could clinically be a better option over p-ACEIs since they are better tolerated by hypertensive patients in the long term. However, this needs further assessment in a larger sample of patients.

The incidence of hypotension with the use of ACEIs is reported to be in the range of 2–5% and there is no significant difference in the incidence of hypotension caused by d-ACEIs or by p-ACEIs.[7,9,13,14] However, in our study, hypotension was observed only in a few subjects receiving fosinopril that needed reduction in dosage from 10 mg/day to 5 mg/day. This feature was observed approximately after a month of starting therapy with fosinopril. On the other hand, none of the subjects in the other study group receiving enalapril, lisinopril, and ramipril had hypotension. As our sample size was relatively small, further studies are required to confirm whether the incidence of hypotension is higher with fosinopril (p-ACEIs) and negligible with enalapril, lisinopril, and ramipril (d-ACEIs). This could also probably mean that the doses were well titrated against the response in terms of the beneficial effect on blood pressure.

Nausea with use of ACEIs is around 1–5% as reported in the literature.[7,9,13,15] It is somewhat higher with lisinopril as compared to enalapril and ramipril[7,13,15] and considerably low with fosinopril.[9] Our findings indicated that the incidence of nausea was higher (10%) with all three agents, namely, enalapril, lisinopril, and ramipril (d-ACEIs), whereas, none receiving fosinopril (p-ACEI) had nausea. From the point of view of patient compliance, p-ACEI fosinopril appears to be a better option, if patients complain of nausea with d-ACEIs. This kind of an increased incidence and severity of nausea with di-carboxyl containing ACE Inhibitors, may at some point be noted by clinicians with caution (although not serious), while prescribing them. In such situations a phosphonate containing ACE Inhibitor may prove a suitable option. The causality needs to be confirmed by evaluating a larger number of subjects to make the study representative of the Indian population.

Based on the observations, it can be concluded that three ADRs, namely, cough, hypotension, and nausea seem to have a probable relationship with the functional group (di-carboxyl / phosphonate). Cough and hypotension have a higher incidence and severity with p-ACEIs, whereas, nausea is associated only with d-ACEIs.

## Limitations of this study

Financial and time constraints not only restricted the number of subjects recruited in our study, but also the duration of the follow-up period and the number of follow-up visits. As this study was limited to 120 subjects, incorporating more number of subjects in each group and conducting the study in different centers across India can establish a more accurate causal relationship. There is a need for an increased number of follow-up visits, over a longer duration, to make this study broad-based and representative of the Indian population.

## Scope for further work

Comparative studies which focus on the association of ADRs with a change in the structure of ACE Inhibitors; involving all the three zinc-binding groups of ACE Inhibitors, namely, sulfhydryl, di-carboxyl, and phosphonate are needed. A phosphonate group replacing the di-carboxyl group was introduced with the intention to increase the potency of ACE Inhibitors. Further studies can be carried out by introducing a newer zinc-binding group in the ACE Inhibitors which may help in discovering a prototype or an ideal ACE Inhibitor having a favourable ADR profile and good tolerability.
